# Patients-centered SurvivorShIp care plan after Cancer treatments based on Big Data and Artificial Intelligence technologies (PERSIST): a multicenter study protocol to evaluate efficacy of digital tools supporting cancer survivors

**DOI:** 10.1186/s12911-021-01603-w

**Published:** 2021-08-14

**Authors:** Izidor Mlakar, Simon Lin, Ilona Aleksandraviča, Krista Arcimoviča, Jānis Eglītis, Mārcis Leja, Ángel Salgado Barreira, Jesús G. Gómez, Mercedes Salgado, Jesús G. Mata, Doroteja Batorek, Matej Horvat, Maja Molan, Maja Ravnik, Jean-François Kaux, Valérie Bleret, Catherine Loly, Didier Maquet, Elena Sartini, Urška Smrke

**Affiliations:** 1 Faculty of Electrical Engineering and Computer Science, University of Maribor, Koroška cesta 46, 2000 Maribor, Slovenia; 2 Institute of Clinical and Preventive Medicine of the University of Latvia, Riga, Latvia; 3 Riga East Clinical University Hospital, Riga, Latvia; 4 SERGAS - Galician Healthcare Service, Galicia, Spain; 5 Univerzitetni Klinicni Center Maribor, Maribor, Slovenia; 6 Data Science Department, Symptoma, Vienna, Austria; 7 Department of Internal Medicine, Paracelsus Medical University, Salzburg, Austria; 8 Physical and Rehabilitation Medicine Department, Centre Hospitalier Universitaire de Liège, Université de Liège, Liege, Belgium; 9 Service of Sénologie, Centre Hospitalier Universitaire de Liège, Liege, Belgium; 10 Department of Gastroenterology, Centre Hospitalier Universitaire de Liège, Liège, Belgium; 11 CyberEthicsLab, Rome, Italy

**Keywords:** Cancer survivors, Well-being, Quality of life, Single-case experimental prospective study, Digital intervention, Artificial intelligence, Health application, Conversational agent

## Abstract

**Background:**

It is encouraging to see a substantial increase in individuals surviving cancer. Even more so since most of them will have a positive effect on society by returning to work. However, many cancer survivors have unmet needs, especially when it comes to improving their quality of life (QoL). Only few survivors are able to meet all of the recommendations regarding well-being and there is a body of evidence that cancer survivors’ needs often remain neglected from health policy and national cancer control plans. This increases the impact of inequalities in cancer care and adds a dangerous component to it. The inequalities affect the individual survivor, their career, along with their relatives and society as a whole. The current study will evaluate the impact of the use of big data analytics and artificial intelligence on the self-efficacy of participants following intervention supported by digital tools. The secondary endpoints include evaluation of the impact of patient trajectories (from retrospective data) and patient gathered health data on prediction and improved intervention against possible secondary disease or negative outcomes (e.g. late toxicities, fatal events).

**Methods/design:**

The study is designed as a single-case experimental prospective study where each individual serves as its own control group with basal measurements obtained at the recruitment and subsequent measurements performed every 6 months during follow ups. The measurement will involve CASE-cancer, Patient Activation Measure and System Usability Scale. The study will involve 160 survivors (80 survivors of Breast Cancer and 80 survivors of Colorectal Cancer) from four countries, Belgium, Latvia, Slovenia, and Spain. The intervention will be implemented via a digital tool (mHealthApplication), collecting objective biomarkers (vital signs) and subjective biomarkers (PROs) with the support of a (embodied) conversational agent. Additionally, the Clinical Decision Support system (CDSS), including visualization of cohorts and trajectories will enable oncologists to personalize treatment for an efficient care plan and follow-up management.

**Discussion:**

We expect that cancer survivors will significantly increase their self-efficacy following the personalized intervention supported by the m-HealthApplication compared to control measurements at recruitment. We expect to observe improvement in healthy habits, disease self-management and self-perceived QoL.

*Trial registration* ISRCTN97617326. https://doi.org/10.1186/ISRCTN97617326. Original Registration Date: 26/03/2021.

## Background

Cancer is the second cause of mortality worldwide, with 18 million new cases in 2018 and 9.5 million deaths [1, 2]. A significant increase of new cases is expected in the next 20 to 40 years. In 2018, 3.91 million new cases of cancer (excluding non-melanoma skin cancer) were estimated in Europe [3], including approximately 1.6 million patients of working age [4, 5]. The most common cancer sites were breast (523,000 cases), followed by colorectum (500,000) [3]. The numbers of cancer surviors are increasing due to advances in cancer treatment and early detection are. In the EUROCARE study [6], the 1999–2007 European mean age-standardized 5-year relative survival for breast cancer (women only) was 81.8% (95% CI 81.6–82.0). For colorectal cancer, the European mean age-standardized 5-year survival was 57.0% (95% CI 56.8–57.3). More than half of the European cancer patients survive 5 years or longer after diagnosis leading to more cancer survivors who experience long-term or latent side effects as a result of cancer treatments [6].

It is very encouraging to see substantially more people surviving cancer, especially as many of them will have a positive effect on society. However, many cancer survivors have unmet needs, especially when it comes to improving the quality of life (QoL), in addition to lengthening of life span [7]. Study in [8] showed that approximately one in four survivors face moderate to severe physical or psychological issues after their cancer treatment. Some cancer survivors have levels of fatigue threefold greater than the general population [9], and many carry a life-long fear of cancer recurrence [10]. Cancer survivors from vulnerable populations or with a low-socioeconomic status are particularly at risk of having QoL lower as compared to the general population [11]. Returning to work had been observed as difficult for a significant number of survivors, A survey in [12] reports that over one-third of employers highlighted concerns about workplace discrimination against cancer survivors.

Fear of cancer recurrence, late toxicity limitations, comorbidities, nutritional disorders and high levels of psychological distress and depression, including the risk of suicide, are generally not appropriately assessed in the guidelines’ recommendations used in follow-up after treatment. However, scientific publications in recent years reflect the growing interest in evaluating and preventing these and other problems to improve the quality of life of cancer survivors.

In the last decades, evidence is emerging that lifestyle of cancer survivors positively influence cancer prognosis as for example, exercise [13], increased fruit and vegetable consumption, healthy body weight and body composition [14], smoking cessation [15], and cognitive behavioral therapy [9]. However, only few survivors can follow all of these recommendations [15]. There is significant research highlighting how the health policy and national cancer plans often neglected cancer survivors’ actual needs [16]. Furthermore, the increasing number of cancer survivors is likely to have a substantial economic impact on the health system.

The PERSIST European Project: "Patients-centered SurvivorShIp care plan after Cancer treatments based on Big Data and Artificial Intelligence technologies", was developed to improve health outcomes, QoL and promote stress reduction related to breast and colorectal cancer survivors, who have gone beyond curative cancer treatment. The choice of these two cancers was based on their relatively high incidence and survival rates, constituting a large number of survivors whose follow-up can be improved.

PERSIST consortium aims at developing an open and interoperable ecosystem to improve the care of cancer survivors. In the project, we expect to achieve increased self-efficacy and satisfaction with care. We also foresee a reduction in psychological distress due to better management of the consequences of cancer and treatment. Compared to usual care, PERSIST interventions are expected to (1) improve health and well-being and, where applicable, contribute to faster reintegration into the labor market, (2) to increase the effectiveness of cancer treatment and follow-up by supporting the decision-making with prediction models, trained on Big Data (3) contribute to more optimal treatment decisions that will reflect in positive (HR)QoL outcomes of survivors, and (4) improve information and evidence to increase the efficacy of management, intervention and prevention policies targeting timely treatment of side effects and, if possible, avoidance of secondary diseases and fatal events.

Several studies have shown how patients and survivors can benefit from digital tools and specifically, solutions that capture patient-reported outcomes (PROs) [17, 18] or solutions that capture patient gathered health data [19]. Nevertheless, the integration of digital tools in clinical settings still raises several different questions. The clinical study is specifically contemplated to assess the efficacy of patient interactions with a mobile device, the quality of data reported by patients using the mHeathApp, and data artificially classified using complex software sensors (i.e., mood estimation, recognition of symptoms of depression). A detailed understanding of different individual, intervention and design factors that have the potential to alter quality of collected data is needed [20, 21].

### Objectives and hypotheses

The objective is to determine if and how a mobile health system (mHealthApp), supported by data-driven Clinical Decision Support System (CDSS), both developed within the project PERSIST [22], will positively affect the behavior of survivors of breast cancer and colorectal cancer. The PERSIST intervention is expected to increase survivors’ self-efficacy and satisfaction with care as well as to reduce psychological stress for a better management of the consequences of cancer and its treatment. Intervention should result in an improved health outcomes and well-being. It should also re-integrate survivors into the labor market faster. By delivering prediction models from Big Health Data and a tool to support decision-making, PERSIST will enable clinicians to make optimal treatment decisions that have a positive impact on the survivor’s quality of life and health. The improved information and evidence will increase the effectiveness in cancer treatment and follow-up, i.e. effectiveness of management, intervention and prevention policies/strategies in order to respond quickly to the side effects and, if possible, avoid secondary diseases and fatal events, compared to regular routine. The long-term result is expected to reduce the socio-economic burden related to cancer survivors’ care.

The primary clinical objective is to assess the acceptability and usability of the mHealthApp and its impact on perceived self-efficacy and satisfaction with care from the perspective of the survivor. To evaluate the primary objective, two validated tools measuring self-efficacy can be utilized, Communication and Attitudinal Self-Efficacy scale for cancer (CASE-cancer) [23] and Strengths Self-Efficacy Scale (SSES) [24]. CASE-cancer was chosen since self-efficacy has been shown to be a protective effect for survivors who have higher perceived risk of recurrence [25–27]. Indivduals who are at higher risk with respect to health and well-being indicators may benefit more from the digital intervention.

The secondary clinical objectives include measuring the patient activation and acceptance of the mHealthApp and experience of its use among them. Patient activation represents the knowledge, skills, and confidence to manage one's health. In multiple chronic conditions, it has been shown to contribute to improved self‐management behaviors [28, 29]. More active patients are more likely to believe their treatment plans reflect their values, more likely to face side effects, and more likely to initiate a healthier diet after diagnosis than less active patients [30]. Less active patients are less likely to understand their diagnosis, follow treatment regimens, and be satisfied with their care. System Usability Scale (SUS) [31], and User Experience Questionnaire (UEQ) [32] will be used to assess acceptance, user experience and possible issues regarding the use of mHealth App. Factors with significant impact on behavioral intention and motivation of use of mHealth Apps are perceived usefulness and ease of use, perceived risk and trust, the subjective norm and attitude [33]. Perceived ease of use is especially significant factor for middle-aged and older survivors.

Finally, the secondary clinical objectives also include the evaluation of the effectiveness of health monitoring and patient gathered health data (PGHD). PGHD includes self-reported health and treatment histories, patient-reported outcomes (PROs), and biometric data [34]. There is a growing interest in PGHD in oncology. Namely, patient reported symptomatology tends to provide a significantly richer information source then Clinicians’ ratings of adverse events. Moreover, there is increasing evidence that integrating PGHD into clinical care can improve outcomes over standard care [35]. Despite the abundance of opportunities, the uptake of digitals tools is surprisingly slow. The two major concerns are the interpretation and quality of data and the concern that reliance on PGHD from wearable devices could exacerbate health disparities, especially in elderly and individuals with less-favorable socioeconomic situations [35–40]. Especially in PROs, the quality of self-reported data depends upon respondents’ ability and willingness to provide accurate and valid responses. Inattentive or careless is defined as an action in which respondents provide answers for the sake of the survey (i.e., a form of survey “satisficing”.) [40]. Thus the respondent does not fully read and/or interpret the question and fails to generate a meaningful response. To measure possible inattentive and carless in responding, we will deploy the Directed Questions Scale—DQS [41].

#### Hypothesis

Performing a comparison between the beginning and the end of the intervention, participants will significantly increase their self-efficacy following the personalized intervention supported by the mHealthApp.

#### Primary and secondary endpoints

The primary endpoint is defined as: *increased perceived self-efficacy in participants (80 colorectal, 80 breast cancer survivors from the 4 pilot hospitals) following the mHealthApp supported intervention as measured using CASE-cancer scale for cancer as the validated standardized measure.*

The secondary endpoints include:

- observing modifications in patient activation levels measured using the PAM,
- observing modification in User acceptance depending on the level of support measured via SUS and UEQ,
- description of negative outcomes over time: hospitalization, exacerbations, treatment adherence, depression, recurrence, drug escalation,
- impact of variable modes of delivery (e.g., conversational agent) on quality of answers measure with DQS,
- the impact of patient trajectories (extracted from retrospective data) and PGHD on prediction of possible secondary disease or negative outcome,
- the impact of patient trajectories (extracted from retrospective data) and PGDH on improved intervention against the appearance of secondary diseases, worsening late toxicities or development of fatal events (sudden death, suicide).

## Methods/design

### Study design

**Fig. 1:**
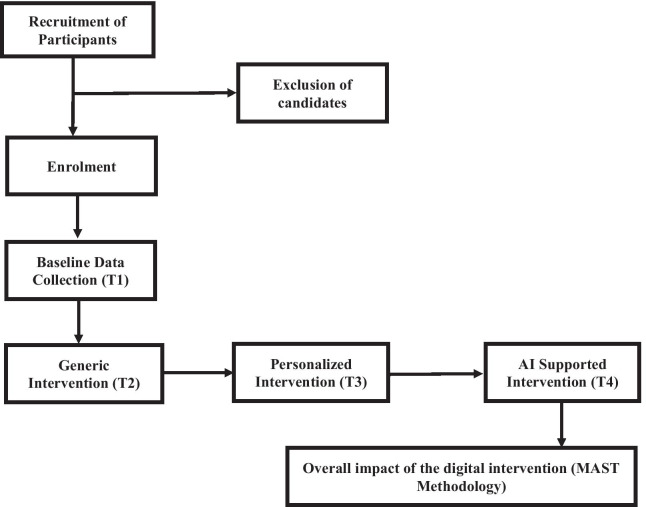
The design of the single-case experimental prospective study

A single-case experimental prospective study was designed in alignment with single-case experimental design (SCED) methodology [42]. Within SCED, each individual is his/her own control group based on the first measurement prior to the intervention. The SCED was selected since single-case designs can capitalize on the ability of technology to easily, unobtrusively, and repeatedly assess health-related behavior [43]. The study design is highlighted in Fig. 1. It is based on the Changing Criterion design in which the intervention and delivery of intervention are modified through personalization of (a) goals/tasks and (b) with spoken language interfaces and embodied conversational agents to complement the regular baseline delivery mode via the digital interfaces of the mHealthApp. The study will take place between 1st of May 2021 and 31st of December 2022, with the recruitment period between 1st of April 2021 and 15th May 2021. Data collection is first carried out prior to intervention, during recruitment round (T1). Each recruited individual will sign a letter of consent and fill in CASE-cancer and PAM questionnaires. The SUS questionnaire responses, along with CASE-Cancer and PAM will be collected following a baseline phase (T2) which will last for 6 months. During this phase a ‘generic’ intervention will include a recording of the diary (at least 3 times a week), report regarding their emotional state (at least once a day) and answering a specific PRO for reporting Health Related QoL and symptoms (a different PRO each day). In total, we expect 10–15 min of interaction every day. T2 will conclude with the first follow-up with clinician, during which data regarding primary (CASE-Cancer, PAM, SUS/EUQ) and secondary endpoints will be collected. The personalized intervention phase (T3) will include execution of tasks on mHealth App personalized for the survivor. The data collection tools will remain the same, however the responsible oncologist will define which of the tools the patient must implement (e.g., which PROs) and in what frequency, depending on the risk assessment implemented by the clinical staff. During intervention, specific tools may change depending on the risks detected for the specific patient. T3 concludes with a follow-up during which data regarding primary/secondary endpoints (CASE-Cancer, PAM, SUS/UEQ). A first comparison between generic intervention and personalized intervention will be made. The AI supported clinical intervention (T4) will introduce embodied conversational agent EVA [44] and AI-drive risk assessment engine to deliver fully personalized intervention and to demonstrate that the data-driven models developed within PERSIST can improve quality of life of cancer survivors by acquiring, managing, sharing, and processing big data to create overall actionable insights at the point of care.

During the final follow-up, the data regarding the primary/secondary endpoints will be collected. To measure the overall impact of the digital intervention we will adapt the Model for the Assessment of Telemedicine (MAST) methodology [45]. The patient domain will be assessed through CASE-cancer, PAM, SUS, UEQ. The clinical relevance will be assessed by: (i) analyzing the impact of Big Data prediction models to support decision-making in patient follow-up, and (ii) the impact of the intervention on against the appearance of secondary diseases, worsening late toxicities or development of fatal events (sudden death, suicide).

#### Study setting

The study will involve 160 survivors (80 survivors of Breast Cancer and 80 survivors of Colorectal Cancer) from four countries, Belgium, Latvia, Slovenia, and Spain. Each of the sites will recruit 40 cancer survivors according to the inclusion/exclusion criteria. Study will involve the use of digital intervention during every-day activities outside the clinical environment (i.e., at the subjects’ homes). The data collected will be evaluated during regular 6 month follow ups.

#### Study itinerary

Follow-up data will include updated information on main outcomes (acceptability and usability). Specific questionnaires related with satisfaction and usefulness will be applied to patients and physicians. In addition, healthy habits, disease knowledge, self-management, QoL, and morbidity and mortality end-points will be analyzed.

The recruited patients will receive the necessary devices for the correct development of the follow-up phase of the clinical study: a smartphone and a wearable quantifying device for physical activity estimation and health parameters measurement (such as blood pressure and heart rate). The overall Implementation of the study is visualized in Table 1.

**Table 1:** The itinerary of the single-case experimental prospective study

Study procedures	Screening/recruitment	Baseline collections (T1)	Follow up visits		
			Visit 2 (T2)	Visit 3 (T3)	Visit 4 (T4)
Informed consent	X				
Inclusion/exclusion criteria	X				
Questionnaires for Primary and Secondary endpoints		X	X	X	X
Medical history (data collection)		X			
Candidate receives a smartwatch	X				
Candidate receives a smartphone	X				

### Participants and recruitment

A 3-month period of recruitment is initially estimated, with 1 month of active enrolment. Before the enrolment, the first 2 months of the period will include a medical evaluation. This will confirm the curative cancer treatment process and verify that the patient meets all inclusion criteria but none of the exclusion ones. During recruitment, we will also provide further information about the study to eligible patients and give them the informative brochure and the informed consent form to sign.

#### Patient information and informed consent

Clinical partners will develop an informed consent document translated in their local language and will apply for their local or national bioethics committee approval. GDPR regulation will be strictly followed to protect personal data of participants. Each center will be responsible to obtain a signed informed consent from the patient before inclusion in the study. The participants will be recruited by 4 hospitals involved in the study from different EU countries (Belgium, Latvia, Slovenia and Spain). Every clinical partner will involve 40 survivors, 20 patients from C18/C19 (Colorectal cancer) and 20 patients from C50 (breast cancer). In total 160 patients will be recruited to participate in envisioned two-phases clinical study. The recruitment stage will begin in April, 2021. The study will involve breast and colorectal cancer patients who have survived beyond curative cancer treatment. We will consider a survivor patient all breast and colorectal cancer patients who survive without recurrence beyond 3–24 months after the end of treatment (surgery ± radiation therapy ± chemotherapy), they have received. For colorectal cancer survivors' group, two subgroups will be included (chemotherapy and non-chemotherapy). None of the groups will be lower than 33% in ratio to the other. For breast cancer survivorspatients that have had surgery and patients that have had chemotherapy.

#### Inclusion criteria

≥ 18 and ≤ 75 years at the moment of recruitment; stable clinical situation, life expectancy of more than 2 years according to researcher opinion; ability to understand study instructions, fulfil follow-up visits and sign informed consent; sufficient level of technology literacy enabling the patient to manage mobile terminals (smartphones, smartphone apps, tablets); good internet connection in his/her place of residence or mobile data-plan.

#### Exclusion criteria

Life expectancy, under the physician opinion, of less than one year; diagnosis of dementia or cognitive decline that makes him/her unable to understand study information and/or sign informed consent; low capacity for self-management due to dependence on other person for medication compliance, or measuring blood pressure and daily weigh; lack of decision capacity in relation with diet or preparing meals; current participation in other clinical study; patient has no further follow-up possibilities with enrolling investigation during planned study period (such as anticipated relocation); patients with major depression and/or psychiatric medication that hinders their daily activity.

### Intervention design

#### Training sessions

The intervention will be delivered via mHealthApp and a smart band. The data related specifically to the primary and secondary end-point will be collected during regular follow-ups. During recruitment, patients will receive a smartphone the smart band and training materials. Demonstration workshops (at some hospitals) will be organized to showcase the use of the mHealthApp and resolve possible initial issues. During the intervention, technical support will be available to resolve any possible complications.

#### Possible HRQoL specific measures

##### General assessment (functioning and self-efficacy) and recurrence

All cancer survivors should be assessed at least once a year. The assessment should include symptoms related to cancer and symptoms prior to treatment. The appropriate follow-up care should be clinically indicated. This assessment can be done by the oncologist or primary care clinician.

Depending on the cancer type and stage of disease, transition of care to primary care may be done when deemed clinically appropriate, with referral back to oncologic care as needed. It is not assumed all survivorship issues are addressed at every visit. Functioning and disability are concepts in increasing use in clinical settings and in public health and much related to cancer survivorship [46]. The use of functioning as a third health indicator could offer much more than the frequency of a disease and the survival rates. From the public health perspective, it reveals information on how the population performs its activities and participation. Validated tools used to screen for long-term and late physical and psychosocial effects of cancer and its treatment survivors already exists. WHODAS 2.0 [47] is a tool to for evaluating health and functioning based on the ICF functional model. It is an alternative to the ICF whose main criticism relates to the effort required to complete. It is 36-item self-administered questionnaire (or proxy-administered) regarding disability. The WHO-DAS II measures disability in 6 domains: understanding and communicating (cognitive), getting around (mobility), self-care, getting along with people, life activities (domestic responsibilities, leisure, work), and participation in society. In addition to 36 item versions, there is also a 12-item version. It is useful for brief assessments of overall functioning in surveys [48].

The General Self-efficacy Scale (GSES) [49] is a validated self-report measure that assesses the overall impression of one’s ability to deal with demanding situations. GSES consists of 10 questions that require patients to self-evaluate, on a scale from 1 (not at all true) to 4 (exactly true), statements regarding their confidence in dealing with unexpected events [50].

Regarding screening, breast cancer imaging with annual mammography and possibly magnetic resonance imaging (MRI) is suggested for women who have been treated with RT. For those not treated with RT, age-appropriate screening guidelines should be followed. Following the NCCN2020 guidelines [37, 51] a PREM was designed as a screening tool focusing on breast cancer recurrence or new primary breast cancer. For survivors treated with RT to the abdomen, pelvis, spine, or traumatic brain injury, COG guidelines recommend surveillance with colonoscopy from age 30, with further interval screening based on clinical findings at colonoscopy.

##### Assessment of Health Related Quality of Life (HRQoL)

QoL represents a person’s perception on their position in life. It reflects the context of the culture and value system in relation to their goals, standards, and concerns. In order to assess HRQoL the European Organization for Research and Treatment of Cancer Core Cancer Quality of Life Questionnaire (EORTC QLQ-C30) [52] was selected as the relevant tool. The EORTC QLQ‐C30 is a 30‐item cancer‐specific HRQoL instrument producing a global health status, five functioning scales (physical, role, social, emotional and cognitive functions), three symptom scales (fatigue, nausea/vomiting and pain) and six single‐symptom items (dyspnoea, insomnia, appetite loss, constipation, diarrhoea and financial difficulties), but no single‐index values.

##### Assessment of cardiovascular risk

Cancer treatments can result in diverse cardiovascular issues. NCCN2020 guidelines [37, 51] focus specifically on heart failure or cardiomyopathy that may arise from anthracycline therapy. However, cardiomyopathy may be laso caused by other systemic therapies, i.e., HER2-targeted therapies and other myopathies, i.e., myocarditis [53]. Signs of cardiac dysfunction can be observed even prior to the development of symptoms [37]. If detected early the cardioprotective medications can mitigate the anthracycline-induced heart failure. However, there is only limited number of prospective studies evaluating these medications [37].

##### Assessment of lymphedema

Lymphedema, a side effect of cancer treatment, results from damage to the lymphatic system. It occurs on the same side of the body as the cancer treatment, when lymph fluid accumulates in the interstitial tissue [37, 51]. This causes swelling of the limb or other areas such as the neck, trunk, or genitals. The diagnosis of lymphedema is, in most cases, confirmed within 18 months of treatment; however, it can develop at any time in the survivor’s life [37, 51]. The cohort with higher risk of developing the lymphedema is represented by survivors that underwent surgery and/or radiation to the axillary, supraclavicular, cervical, or inguinal lymph node system [37, 51, 54]. Since stages 0 and 1 are reversible, early diagnosis is the key to optimal treatment. The stages 2 and 3, however, are less responsive to treatment. The prospective surveillance model (PSM) had been recognized as an optimal framework to guide clinical implementation of a screening methodology for early identification and management of breast cancer treatment-related impairments [55]. PSM can help in early identification and treatment of lymphedema, even in subclinical stage. In the subclinical stage, the intervention can prevent the progression to a more chronic form. According to NCCN2020 guidelines, survivors with high risk; i.e. a history of radiation or surgery to the lymph nodes, should be questioned about swelling or feeling of heaviness, fatigue, or fullness at each visit [37, 51].

##### Assessment of pain

Over one-third of post-treatment cancer survivors experience chronic pain. It often leads to psychological distress; decreased activity, motivation, and personal interactions; and an overall poor quality of life [56]. In general pain can be nociceptive or neuropathic. Somatic nociceptive pain is described as sharp, throbbing, or pressure-like. It often occurs after surgical procedures. The visceral nociceptive pain is often diffuse and described as aching or cramping [56]. Neuropathic pain is a result of an injury to the peripheral nervous system or CNS and might be described as numbness or as burning, sharp, tingling, prickling, electrical, or shooting pain. Often it develops as a side effect of chemotherapy or radiation therapy or is caused by surgical injury to the nerves. For this reason, it is very common among breast and colorectal cancer survivors. Unfortunately, there is few data on effective pharmacological treatments of CIPN.

The NCCN2020 guidelines [37, 51] make recommendations to manage the following seven categories of pain: neuropathic, chronic, myalgias/arthralgias, skeletal, myofascial pain, gastrointestinal/urinary/pelvic pain, and post-radiation pain [37]. It, however, does not include The recommendations regarding prevention and management of chemotherapy-induced peripheral neuropathy (CIPN). The recommendations for CIPN can be found in ASCO’s clinical practice guidelines [57]. Additionally, ASCO provides clinical practice guidelines for the management of chronic pain in adult cancer survivors [58]. In PERSIST the Wong-Baker FACES Pain Rating Scale [59]. It is a unidimensional intensity scale and includes the 11-point numeric rating scale from 0 (no pain) to 10 (extreme pain) accompanied with visual/word descriptors.

##### Assessment of hormonal imbalances

The NCCN2020 guidelines [51] define menopause as: (i) no menses for one year in the absence of prior chemotherapy or tamoxifen use or (ii) no menses after surgical removal of all ovarian tissue [37]. However, significant number of cancer survivors experience menopausal symptoms without meeting the above criteria. In fact, hormonal symptoms in cancer survivors have been most extensively studied in female survivors after treatment of breast cancer. Studies report that hot flashes, vaginal dryness and dyspareunia are common among breast cancer survivors [60]. Other symptoms of hormonal imbalance may be sexual dysfunction, depression, fatigue, etc. [37] From the NCCN2020 guidelines [51] we select questions targeting vaginal dryness, and urogenital complaints associated with menopause when designing the specific PREM. Other symptoms are already addressed in other measures.

##### Assessment of sexual dysfunctions

Sexual dysfunction is often caused by hormonal therapy and surgical or radiation therapy directed towards the pelvis (i.e. during colorectal cancer treatment) [37]. Depression and anxiety are commonly associated with survivorship and can further contribute to imparied sexual function [51]. Sexual dysfunctions after cancer treatment are more common in female survivors [61]. The female dysfunctions relate to issues with desire, arousal, orgasm, and pain. The problems vary depending on the cancer site and treatment modalities used. A prevalence of chemotherapy-induced menopause had been observed within the cohort of breast cancer survivors that were treated using chemotherapy [37]. Furthermore, body image changes related to breast cancer surgery and reconstruction can affect women’s sexual life and well-being [62].

Although effective strategies for treating both female and male sexual dysfunctions exists, sexual function is rarely discussed with survivors. In line with NCCN2020 guidelines [51] and EORTC QLQ-BR45 [63] we designed a 2-question PREM as a screening tool.

##### Assessment of fatigue

In order to promptly diagnose and effectively treat moderate and severe fatigue, all survivors should be screened for symptoms of fatigue [37]. Fatigue is a highly subjective experience. In order to include fatigue in clinical routine the clinicians must understannd patients’ interpretations of their fatigue level. To standardize the patient reports NCCN guideliness propose to evaluate the fatigue through three and one item severity scales [64].

##### Assessment of symptoms of anxiety

Fear of recurrence, distress, anxiety and depression are the most relevant mental health risks for cancer survivors. These risks may persist many years after diagnosis. The NCCN Guidelines provide a questionary for patient self-assessment and an algorithm for oncologists and other health care providers to screen for distress, anxiety, and depression in cancer survivors. The algorithm focuses on common mood disorders after cancer and is not intended as a psychiatric diagnosis tool [37]. For PERSIST the Generalized Anxiety Disorder (GAD-7) [65] questionnaire was chosen to assess symptoms of anxiety. It is a validated diagnosis tool implemented as a 7-item, self-report questionnaire designed to assess the patient’s status during the previous 2 weeks. The items target feelings of nervousness, anxiety, or if the patient was on edge, not being able to stop or control worrying or over-worry different things. It also measures if the patient had trouble relaxing or if patients were easily annoyed or irritable and or afraid.

##### Assessment of symptoms of depression

Symptoms of depression are pervasive in survivors. However, often the distressed survivors may not appear distressed. Therefore, all survivors should be screened for anxiety, depression, and distress, especially at times of disease transition, surveillance, significant loss, major life events, and social isolation [37]. In fact, routine screening of cancer-related distress, including clinical anxiety or depression and post-traumatic stress, should be carried out as part of the standard follow-up of survivors at least annually. Survivors with multiple or repeated somatic complaints should also be screened as part of their overall workup. However, the NCCN guidelines do not address how to detect somatic behaviors.

Patient Health Questionnaire PHQ-9 [66] and Patient Health Questionnaire PHQ-2 [67] were chosen as screening tools. The PHQ-9 is the 9-item depression module. Major depression is suspected if, in the past 2 weeks, five or more of the nine criteria have been observed at least “more than half of the days”. Additionally, one of the symptoms is depressed mood or anhedonia [68]:2. The PHQ-2 is a short version. It uses the first 2 questions of the PHQ-9 to investigate the frequency of the symptoms of depressed mood and anhedonia. Each question is scored as 0 (not at all) to 3 (nearly every day).

##### Assessment of cognitive function

The hypothesis that neurotoxicity resulting in brain white matter damage plays an important role in cognitive deficits after chemotherapy had been supported by multiple studies [69]. Functional MRI studies show that changes in brain activity accompany cognitive complaints or cognitive deficits in survivors [70]. In line with NCCN2020 guidelines [37, 51] and EORTC QLQ-BR45 [62], we designed a 3-question PREM as a screening tool. Further, as a more in-depth tool FACT-Cog [71] can be used to further assess cognitive complaints in cancer patients. FACT-Cog is a 37-item questionnaire. It measures complaints in six cognitive domains: memory, concentration, mental acuity, verbal fluency, functional interference, and multitasking ability. Moreover, the tool includes two other subscales: ‘‘comments from others’’ (i.e. ‘‘noticeability’’) and ‘‘effect of perceived cognitive impairment on quality of life’’.

##### Assessment of healthy lifestyle, nutrition and physical activity

Cancer survivors are often motivated towards self-intervention to improve their treatment outcomes, QoL, and overall survival [72]. They often seek information about dietary options and supplements, and physical activity. Namely, all cancer treatments (i.e., surgery, radiation, and chemotherapy) can significantly affect nutritional habits, alter regular eating patterns, and adversely affect how the body digests, absorbs and uses food. Following NCCN2020 guidelines [51], we designed a PREM for nutritional assessment. It comprises of questions assessing (healthy) nutrients and fluids intake, weight variation, meal frequency, and eating habits.

Further, Physical activity represents an appealing intervention. It could alleviate sequelae related to cancer and assist patients in returning to the health status they had before treatment [73]. It has positive effects in physiology, body composition, physical functions, psychological outcomes, and quality of life in patients after treatment for breast cancer [73]. To assess physical activity the Global Physical Activity Questionnaire (GPAQ) [74] was chosen as the appropriate tool. GPAQ measures the number of minutes spent in three areas, work, leisure, and transportation in a typical week, i.e., spent for walking/cycling to get to and from locations. Using this data GPAQ calculates metabolic equivalents to express the intensity of self-reported physical activities.

#### Data collection

Baseline data to be collected during or right after recruitment (T1): clinical and demographic data as well as social aspects of health status and QoL (social determinants of health). During recruitment baselines, data regarding survivor’s activation (PAM) and self-efficacy (CASE-Cancer) will be collected.

Data collection will be provided by Smart devices LZD Smart band (Fitbit like) from Naicoms and Smartphone. This data includes:

- data reported by patients using the mobile application – based on PROs: we will deliver and select form the following tools: Quality of life for cancer patients (EORTC QLQ‐C30), Patient Health Questionnaire-2 (PHQ-2), Patient Health Questionnaire-9 (PHQ-9), General Anxiety disorder (GAD-7), WHO Disability Assessment Schedule (WHODAS 2.0), proprietary questionnaires were designed by participating oncologists for additional risk assessment related to health-related specific symptoms related to physical symptoms, cardiovascular risks, pain, fatigue, malnutrition, lymphedema, hormonal disbalances, sexual dysfunction, gastrointestinal conditions and cognitive functioning.
- data from the physical activity tracker worn by the patient: Vital signs and physical activity. Blood pressure, heart rate, Blood Oxygen, Steps, Calories, Distance, Running Track, Sleep Monitor—built in the firmware of the watch.
- data from software sensors to be developed by UM and SYM (e.g., facial action units, acoustic features, emotion and mood estimation extracted from diary recordings).

The pseudo-anonymised data will be stored in a Big Data platform hosted in CESGA infrastructures (CESGA is a Spanish public institution offering high performance computing services for administrations and research purposes).

The data is gathered in the following way:

- Manually (T2 and T3)—obtain patient information using questionnaires (PROMs, PREMs and health-related questions) displayed in a Smartphone screen.
- Supported (T4)—the component will converse with the users using natural free speech. It will be able to engage with them in an organic dialogue by helping the user to provide answers to PROs and to health-related questions.
- Automated Classification (T2, T3 and T4): Speech recognition and feature extraction from recorded interaction. Facial biomarkers will be used to extract facial action points. Speech biomarkers will be used to extract acoustic features from the interaction. Text features (i.e., words, sentiment, syntax and semantics, topic and named entity classification and morphology) will be extracted from recognized speech.

### Outcomes (CASE-Cancer, PAM, SUS/EUQ)

The outcomes of this clinical study will be measured and evaluated using CASE-Cancer, PAM, SUS and EUQ. First questionnaire battery (T1) will include CASE-Cancer and PAM, and will be administered in all four batteries (T1–T4) to establish the patients’ baselines. All successive batteries (T2, T3, and T4) will include two additional questionnaires.

The four questionnaire batteries (pre-screening, T1, T2, T3) measure primary outcomes. In the pre-screening questionnaire, additional questions aimed at collecting data on background variables (e.g., demographic data) are foreseen. The questionnaire battery administered at T1, T2, and T3, will also include the patent activation measure. Additional background variables, such as overall satisfaction with the intervention and satisfaction with the system will be assessed at T2 and T3 (directly after the intervention, e.g.,).

### Statistical analysis

Despite this is mainly a pilot study of acceptability, usability and analysis of surrogate variables, sample size is estimated according to expected effectiveness in changing healthy habits in patients. This is based on previous peer-review published studies with mobile devices in cancer survivors.

An article by Pope et al. [75] investigated the impact and feasibility of commercial mobile health applications and social media to improve physical activity and health of breast cancer survivors. They provided a digital health education intervention to breast cancer survivors. The results show positive post intervention trends in increased average daily moderate-to-vigorous physical activity and steps. The results of the study show a notable decrease in weight (2.4 kg) and body fat percentage (2.3%). Quintiliani et al. [76] aimed to evaluate engagement (use and acceptability), physiological (weight), behavioral (diet and physical activity), and other secondary outcomes among Breast Cancer Survivors. The engagement was high: the mean number of days recording steps via the wristband pedometer, recording a weight via scale, and responding to text messages was high, and 100% of participants completed all 4 calls with the counselor. The mean weight of participants decreased. The daily intake of fruit and vegetables increased, and self-reported moderate physical activity, measured in metabolic equivalent of task (MET) minutes per week, increased [76]. Most of the participants would very likely participate again and would recommend the app to others.

#### Sample size calculation

To estimate the sample size to difference between two dependent means we have based on the assumptions of a two-sided confidence level of 95%, a statistical power of 90% and effect size Cohen’s *d* of 0.25. The previous assumptions indicate that 160 patients may detect differences between pre and post variables of healthy habits. A G*Power 3.1 software was used to make this sample size estimation.

#### Statistical analysis

A descriptive analysis of clinical characteristics will be performed. The quantitative parameters will be summarized by its average ± standard deviation and percentiles, while for the qualitative variables the frequencies and percentages will be computed correspondingly.

In order to test the objectives, for CASE-cancer, SUS/UEQ and scores as well as for single items, differences between pre, post, and follow-up measurements will be analyzed by parametric test (Student’s *t*-test/ANOVA), non-parametric test (Wilcoxon signed-rank test) and McNemar test (for categorical measures).

Descriptive statistics of the negative outcomes and/or complications (hospitalization, exacerbations, treatment adherence, depression, reoccurrence and drug escalation) reported in the study will be used. These measures and patient trajectories (appearance of secondary diseases, worsening late toxicities or development of fatal events) will compare with data extracted from retrospective study. We will use univariate tests like Student’s *t*-test and chi-square test and multivariate models. For all the comparisons, sub-analyses will be carried out stratifying by type of cancer, sex and any other variable considered of interest.

An intention-to-treat analysis and sensitivity analysis of observed data will be performed. We will use R 3.4.2 and SPSS version 19 for statistical analysis. Test results with a *p* value below 0.05 will be deemed statistically significant.

## Discussion

Cancer survivors have complex needs that must be fully recognized and addressed in a comprehensive and coordinated manner. In the last decades, evidence is emerging that lifestyle of cancer survivors positively influence cancer prognosis as for example exercise, increased fruit and vegetable consumption, healthy body weight and body composition, smoking cessation, and cognitive behavioral therapy. However, few survivors can meet all of these recommendations. According to the “European Guide on Quality Improvement in Comprehensive Cancer Control” [77], “follow-up, late effect management and tertiary prevention need to be anticipated, personalized and implemented into care pathways, with the active participation of survivors and relatives.” This study also concludes that “no clear consensus exists on follow-up care plans for survivors” and that “more research in survivorship is needed. The research needs to provide data on late effects, and data on impact and cost-effectiveness of supportive care, rehabilitation, palliative and psychosocial care interventions” [77]. The global challenge is to provide evidence base and tools for the development of policy strategies to improve the delivery of cancer survivorship care and the QoL after the cancer treatment (including prevention, early diagnosis, therapies as well as addressing health inequalities). The proposed clinical study targets a demand towards a more patient-centered cancer survivorship care to increase awareness and self-efficacy (engagement in their care plans). To address this demand a mHealth App capable of collecting PGHD and PROs is delivered and evaluated. Often survivors remain unaware of their risk of recurrence and late effects and have no holistic plan for follow-up care. This study is expected to highlight the main benefits of digital interventions (Big Data, Artificial Intelligence and Digital Sensing) as:

- increased self-efficacy and satisfaction with care and reduced psychological distress regarding management of side effects or outcomes of treatment and the disease itself. This will result in an improvement in health and well-being and a faster integration in the labor market, where applicable, compared to usual care;
- increased effectiveness of cancer treatment and follow-up routine supported by Big Data based knowledge models. The models will support decision-making and contribute to optimal treatment decisions. This will have direct impact on the QoL and the health status of survivors;
- improved patient context (i.e. information) and evidence to improve the efficacy of management, intervention and prevention policies/strategies, all targeting timely mitigation of side effects and, if possible, avoidance of secondary diseases and fatal events. The long-term result are foreseen to reduce the socio-economic burden related to cancer survivors’ care.

## Data Availability

Not applicable. No sensitive data will be made available. Specific statistical cohorts will be open to wider public.

## Abbreviations

ANOVA: Analysis of variance
ASCO: American Society of Clinical Oncology
C18/C19: Colorectal cancer
C50: Breast cancer
CASE-cancer: Communication and Attitudinal Self-Efficacy scale for cancer
CDSS: Clinical Decision Support system
CIPN: Chemotherapy-induced peripheral neuropathy
CNS: Central nervous system
DQS: Directed Questions Scale
EORTC QLQ-BR45: European Organization for Research and Treatment of Cancer Core Cancer Quality of Life Questionnaire—module BR45
EORTC QLQ-C30: European Organization for Research and Treatment of Cancer Core Cancer Quality of Life Questionnaire—module C30
FACT-Cog: Functional Assessment of Cancer Therapy—Cognitive Function
GAD: Generalized Anxiety Disorder
GPAQ: Global Physical Activity Questionnaire
GSES: General Self-efficacy Scale
HRQoL: Health Related Quality of Life
ICF: International Classification of Functioning, Disability and Health
MAST: Model for the Assessment of Telemedicine
mHeathApp: Mobile Health Application
MRI: Magnetic resonance imaging
NCCN: National Comprehensive Cancer Network
PAM: Patient Activation Measure
PERSIST: Patients-centered SurvivorShIp care plan after Cancer treatments based on Big Data and Artificial Intelligence technologies
PGHD: Patient Gathered Health Data
PHQ: Patient Health Questionnaire
PREM: Patient-Reported Experience Measure
PRO: Patient reported outcome
PSM: Prospective surveillance model
QoL: Quality of life
SCED: Single-case experimental design
SPSS: Statistical Package for the Social Sciences
SSES: Strengths Self-Efficacy Scale
SUS: System Usability Scale
UEQ: User Experience Questionnaire
WHO: World Health Organization
WHODAS: WHO Disability Assessment Schedule
